# Effect of locoregional surgery in primary tumors on overall survival in patients with *de novo* stage IV breast cancer: a systematic review and meta-analysis

**DOI:** 10.3389/fonc.2025.1590246

**Published:** 2025-05-13

**Authors:** Yanbo Sun, Hao Ma, Yingjie Li, Can Zhou

**Affiliations:** ^1^ Department of Breast Surgery, First Affiliated Hospital of Xi’an Jiaotong University, Xi’an, Shaan’xi, China; ^2^ School of Medicine, Xi’an Jiaotong University Health Science Center, Xi’an, Shaan’xi, China

**Keywords:** locoregional surgery, overall survival, meta-analysis, *de novo* stage IV breast cancer, ER/PR-positive

## Abstract

**Background:**

Due to the controversy in the therapeutic effect of locoregional surgery in primary tumors for patients with de novo stage IV breast cancer, the aim of this study was to evaluate the effect of locoregional surgery on overall survival in patients with de novo stage IV breast cancer.

**Methods:**

A computer-based search of PUBMED, Embase, and American Society of Oncology (ASCO) annual meetings abstracts was conducted to identify the prospective trials of the combination of locoregional surgery in primary tumors and systemic therapy in comparison with standard systemic therapy alone for patients with de novo stage IV breast cancer. Hazard ratios (HR) and 95% confidence intervals (CI) were calculated by universal inverse variance and combined across articles. Random-effects model and subgroup analyses were performed to ascertain the origin of this heterogeneity.

**Results:**

A total of 2029 patients from 8 studies were included, with 1014 patients (49.98%) underwent locoregional surgery in primary tumors (surgery group) and 1015 ones (50.02%) with standard systemic therapy alone (no surgery group). Compared to patients in the no surgery group, participants with ER/PR positive breast cancer in the surgery group had improved overall survival (OS) (HR=0.77, 95%CI 0.55-0.93, P=0.01), and improved locoregional progression-free survival (HR=0.36, 95%CI 0.14-0.95, P=0.04) for all participants in the surgery group. And patients with bone-only metastases in the surgery group had insignificantly favorable OS than those in no surgery group (HR=0.70, 95%CI, 0.47-1.04, P=0.08).

**Conclusion:**

Our study demonstrated that locoregional surgery in primary tumors was associated with improved OS for participants with ER/PR positive *de novo* stage IV breast cancer, and locoregional surgery in primary tumors could be worthy of clinical recommendation for patients with ER/PR positive de novo stage IV breast cancer.

## Introduction

As a common malignant disease in women, approximately 6% of patients with breast cancer had distant metastases at first diagnosis (1–4), known as de novo stage IV disease (5). For such patients, systemic therapy (ST), which was determined by hormone receptor (HR) and HER2 expression status, was always recommended as a first-line treatment option by the current international guidelines (6–8). In the clinical practice, localregional treatment (LRT) (surgery or radiotherapy in primary tumors), were always performed to control tumor-related symptoms for patients with de novo stage IV breast cancer, such as pain, skin ulcers, bleeding, and infection (6, 9), although the controversy (10, 11) in the long term survival benefit of locoregional surgery in primary tumors for patients with de novo stage IV breast cancer.

Many retrospective studies have shown that the use of locoregional surgery in primary tumors could effectively improve the overall survival (OS) for patients with *de novo* stage IV breast cancer (12–18). However, retrospective studies were inherently subject to selection bias and other potential confounders, since younger, healthier patients who responded well to systemic therapy were more likely to undergo surgery (19). In contrast, several prospective randomized controlled trials had produced conflicting results. Many trials reported early locoregional therapy for the primary site did not improve survival in patients presenting with metastatic breast cancer (20–23). However, the MF07-01 trial showed that patients who received LRT followed by ST had a 14% higher OS benefit by the end of the 10-year follow-up compared with the patients who received only ST (16).

To determine the effect of locoregional surgery in primary tumors for *de novo* stage IV breast cancer patients, a comprehensive meta-analysis of prospective trials was performed to summarize the literature and evaluate the impact of surgical treatment in primary tumors on the survival rate of newly diagnosed stage IV breast cancer patients according to molecular subtype and metastatic site.

## Methods

### Search strategy

Several sources for relevant original publications were searched through Pubmed, Embase, American Society of Oncology (ASCO) annual meetings abstracts and other databases from January 1, 2003 to February 1, 2025, by the following terms as key words: (stage IV) or (*de novo*) or (metastatic)) AND (breast cancer) AND ((local) OR (surgery) OR (radiotherapy)), with setting filters: women, prospective trials, and adults. All search strategies were performed in accordance with the Preferred Reporting Items for Systematic Reviews and Meta-Analyses (PRISMA) guidelines (24).

### Inclusion and exclusion criteria

Studies that met the following criteria were included: (1) randomized controlled trials and prospective observational studies comparing the combination of surgery in primary tumors plus systemic therapy with systemic therapy alone, (2) adults initially diagnosed with stage IV breast cancer without prior anticancer therapy, and (3) having appropriate survival data - HR and 95% confidence interval (CI) for overall survival (OS) for patients.

Studies were excluded if: (1) hazard ratios (HRs) or 95% CIs for OS were not reported, or the full text was not available for data extraction; (2) all patients underwent surgical resection of the primary tumor alone; (3) reviews, retrospective studies or meta-analyses; (4) not newly diagnosed stage IV breast cancer. This process was independently screened by two authors (Yanbo Sun and Hao Ma), and disagreements were resolved by the third author (Yingjie Li).

### Data extraction

The data collected included the author's name, publication year, median follow-up time, mean age,the country for the first author, and the number and proportion of patients in the surgery group versus non-surgery group for each tumor characteristic. TNM stage, histological grade, hormone receptor status, HER2 receptor status, molecular subtype, metastatic site, and the number of metastatic sites were also extracted. For the outcome measures, hazard ratio (HR) and 95% confidence interval (CI) were extracted for overall survival (OS), local progression-free survival (LPFS), and distant progression-free survival (DPFS). Survival data according to molecular subtype and metastatic site were also extracted.

Two authors (Yanbo Sun and Hao Ma) separately used the Cochrane Risk of Bias tool to assess the risk of bias in seven domains for each study (25). For each domain, we graded the risk of bias into the following three levels: unclear risk, low risk, and high risk.

### Measures of treatment effect and statistical analysis

OS was used as the primary endpoint, local progression-free survival (LPFS) and distant progression-free survival (DPFS) and hazard ratio (HR) and its 95% confidence interval (95%CI) were performed as the secondary endpoints. At the same time, subgroup analysis was performed to explore the effect of surgery on OS in different subgroups, according to tumor molecular subtype and metastatic site.

Random-effects model with the inverse variance method was performed to obtain summary estimates of RR and 95% CI using Review Manager 5.4 software (Cochrane Collaboration, Oxford, United Kingdom) softwares. The value of HR lower than 1 (<1) was defined as the survival benefit of locoregional surgery in primary tumors, and *P<0.05* was statistically significant. Cochran’s Q test or I^2^ statistic were used to estimate the size of heterogeneity, and *P<0.10* or I_2_ greater than 50% (>50%) was considered the significant heterogeneity. To investigate the source of heterogeneity and the effect of locoregional surgery in primary tumors on OS, subgroup analyses were performed. For the sensitivity analysis, the study were removed one by one and the meta-analysis were-conducted to determine the impact of each study on the meta-analysis. In addition, a funnel plot was performed to assess the risk of publication bias, and forest plots were applied to report the results of the meta-analysis.

## Results

**Figure 1:**
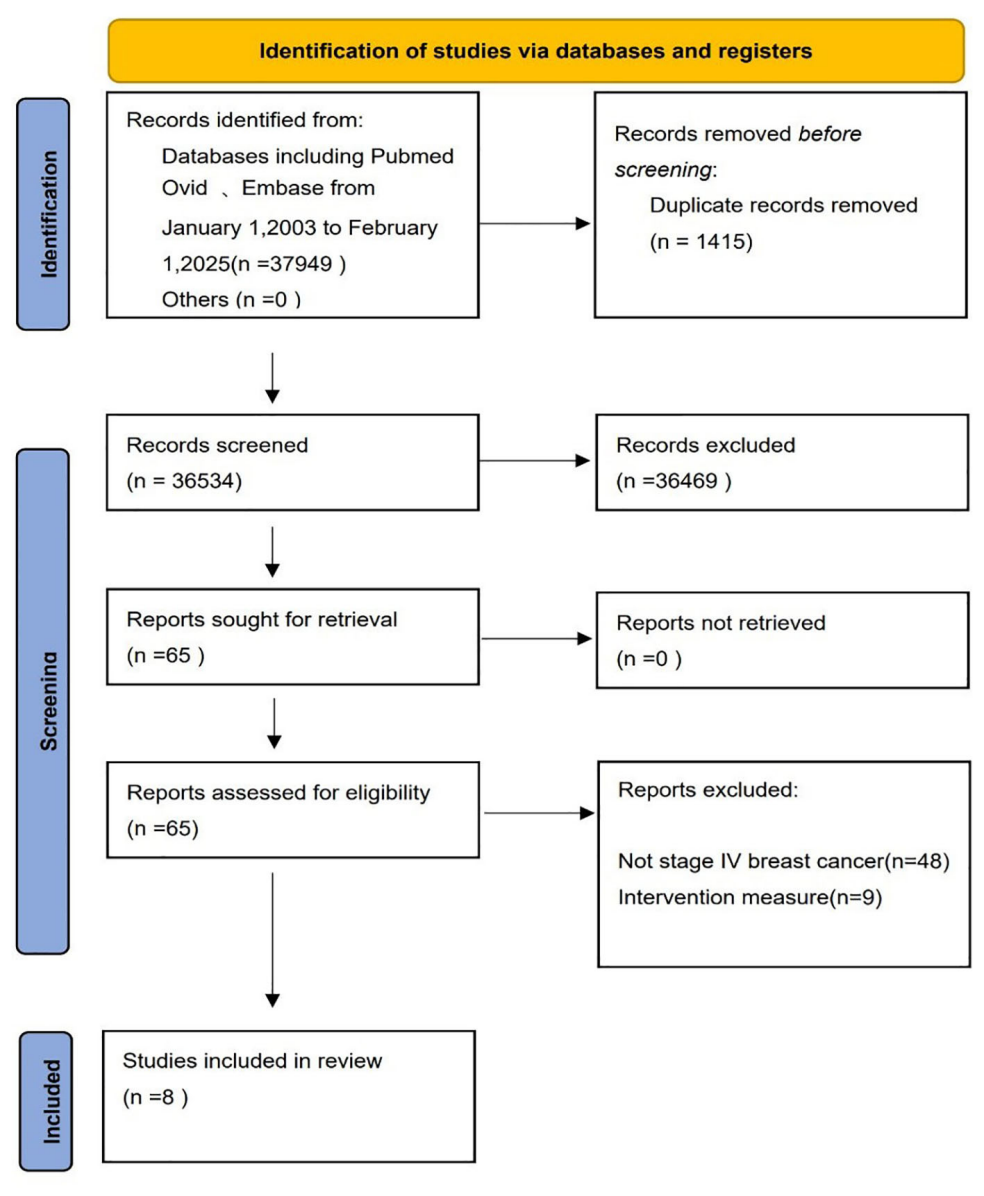
Flow chart of literature screening according to PRISMA guidelines.

First, among the 37,949 articles found according to the search strategy, a total of 36,534 ones were left after removing the 1415 duplicates. After reading the titles and abstracts,and full texts, a total of 8 studies including 5 RCTs and 3 prospective studies and 2029 patients (17, 18, 20–23, 26, 27), were included. Totally, 1014 patients (49.98%) had underwent locoregional surgery in primary tumors (surgery group), and 1015 patients (50.02%) had no surgery (non-surgery group), as shown in **Figure 1** and in **Table 1**. **Table 1** describes the characteristics of the included studies and their patients.

**Table 1:** Characteristics of the included studies in the meta- analysis.

Author	Duration of study	Number of patients	Median follow-up time (months)	ER/PR positive	HER2 positive	Triple negative	Only Bone metastasis	Bone and visceral metastases
Seema. Khan 2022	2011-2019	256	53	57.0%	32.2%	7.8%	41.7%	N/A
Rajendra Badwe 2015	2005-2013	350	23	59.4%	30.6%	N/A	28.5%	28.0%
Florian Fitzal 2019	2011-2015	90	37.5	64.4%	22.2%	8.9%	37.8%	62.2%
Atilla Soran 2018	2007-2012	274	40	78.5%	30.7%	12.0%	46.0%	25.5%
Atilla Soran 2021	2014-2021	505	34	85.1%	28.5%	7.1%	100%	N/A
Abo-Touk 2016	2012-2016	57	15	N/A	N/A	N/A	63.2%	40.4%
TA King 2016	2011-2016	90	54	N/A	29.0%	7.0%	N/A	N/A
Shien. T 2023	2011-2023	407	60	N/A	29.7%	N/A	28.7%	N/A

N/A, not applicable.

### Effect of local surgery on overall survival (OS)

**Figure 2:**
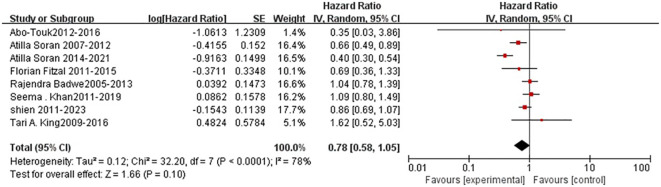
Forest plot: overall survival.

**Figure 3:**
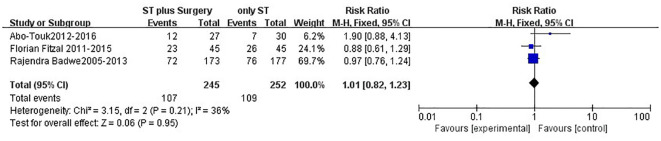
Forest plot: 2-years overall survival.

**Figure 4:**
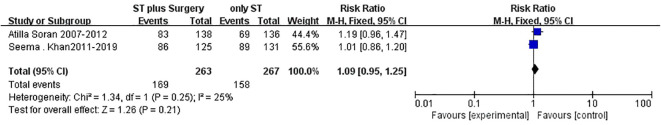
Forest plot: 3-years overall survival.

After a median follow-up time of 40 months, patients in the surgery group had a insignificantly statistically (P=0.10) higher overall survival (OS) rate than those in the non-surgery group (HR=0.75; 95%CI, 0.58-1.05) (**Figure 2**), although there was statistically significant heterogeneity (*P<0.0001*, I^2^=78%). Besides, no benefit of 2-year OS (relative risk [RR] = 1.01; 95 % CI 0.82–1.23, *P = 0.95*; **Figure 3**), or 3-year OS (RR = 1.09; 95 % CI 0.95–1.25, *P = 0.21*; **Figure 4**) were found for patients in the surgery group.

### Effect of local surgery on local progression-free survival and distant progression-free survival

**Figure 5:**
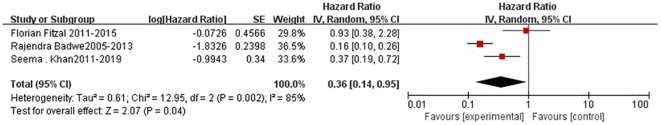
Results of the meta-analysis of local progression-free survival among patients with *de novo* stage IV breast cancer.

**Figure 6:**
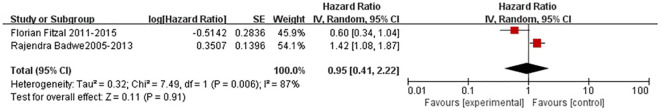
Meta-analysis of distant progression-free survival among patients with *de novo* stage IV breast cancer.

Totally, 3 trials reported HR data on LPFS after primary tumor resection. Patients in the surgery group had significantly (*P=0.04*) improved LPFS than those in the no-surgery group (HR=0.36, 95%CI,0.14-0.95, **Figure 5**), although there was statistically significant heterogeneity among the trials (*P =0.002*, I^2^=85%, **Figure 5**). Moreover, for distant progression-free survival (DPFS), only 2 trials reported the relevant HR data, and the pooled HR of 0.95 (95%CI,0.41-2.22) showed that patients in the surgery group had similar DPFS to those in the non-surgery group (*P=0.91*, **Figure 6**).

### Effect of local surgery on subgroup analysis

**Figure 7:**
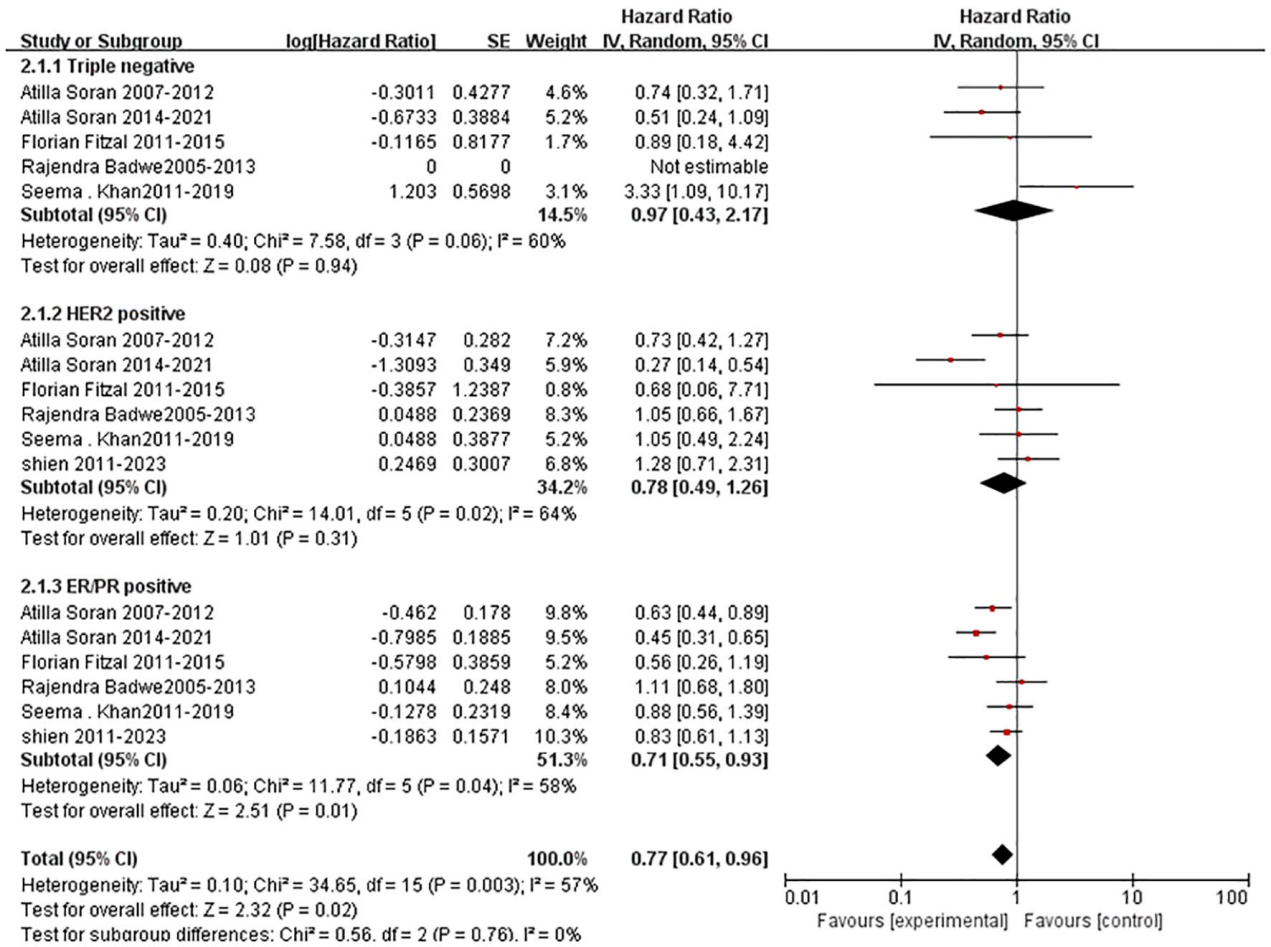
Results of subgroup analysis of overall survival by molecular subtype for surgical versus nonsurgical comparison.

Subgroup analyses were performed according to molecular subtyps and site of metastasis. A total of 6 trials reported the HR data on overall survival (OS) by molecular subtypes. The pooled HR of 0.71 (95%CI,0.55-0.93, **Figure 7**) indicated that the primary tumor resection could significantly statistically improve the overall survival for candidates with ER/PR positive diseases (*P=0.01*, **Figure 7**)., although statistically significant heterogeneity existed among the trials (*P=0.04*, I^2^=58%, Figure 7). However, for triple-negative or HER2-positive diseases, no OS benefit were found after the adminstration of locoregional surgery in primary tumors (**Figure 7**).

**Figure 8:**
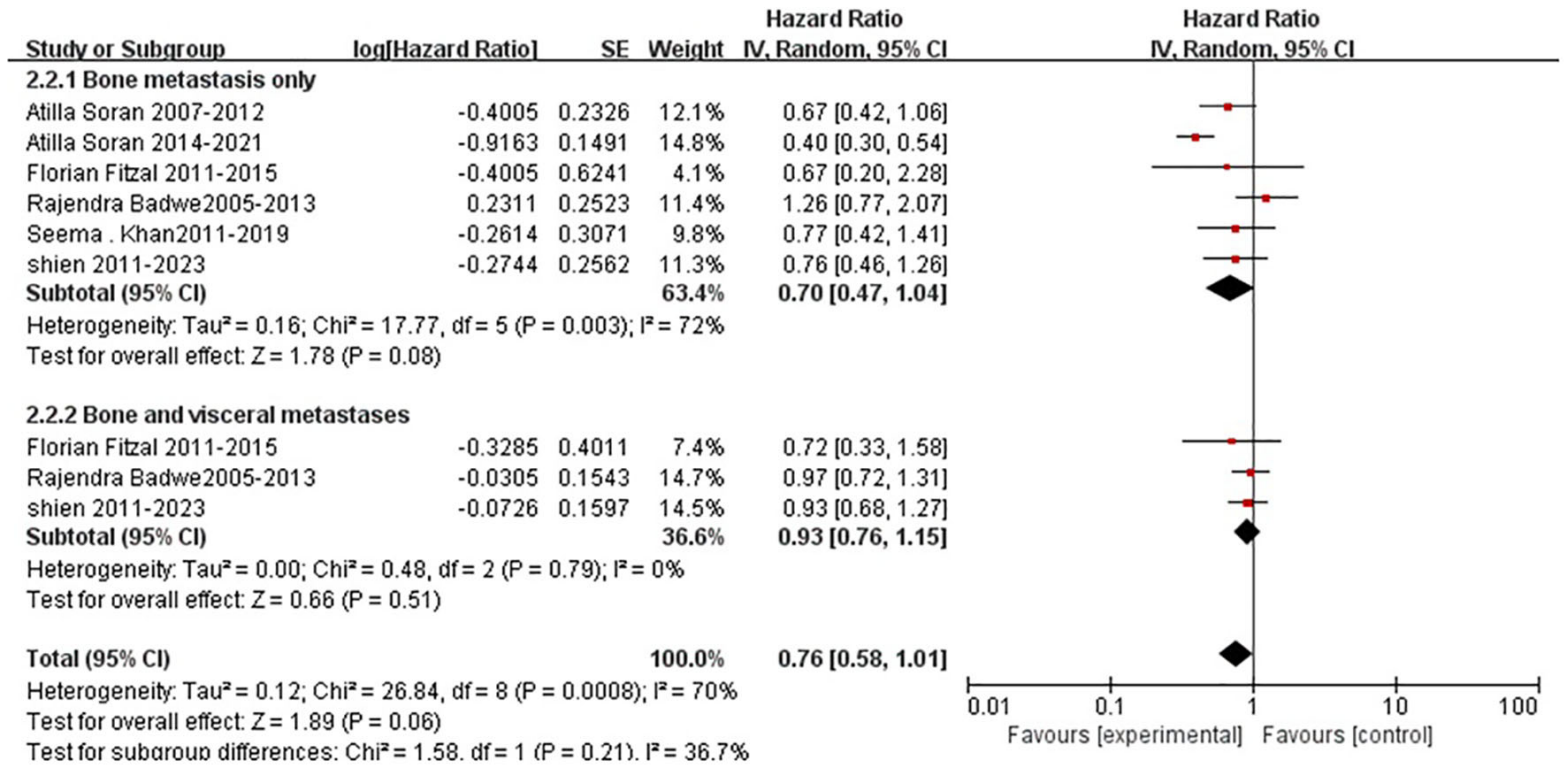
Results of subgroup analysis comparing overall survival of metastatic sites with surgery and without surgery.

Six trials reported the survival data for patients with bone metastases only, and three trials reported the survival data for patients with both bone and visceral metastases. For women with bone-only metastases as well as those with both bone and visceral metastases, patients in the surgery group had statistically insignificantly improved overall survival than those in the no-surgery group (bone-only metastases: HR=0.70, 95%CI,0.47-1.04; Bone and visceral metastasis: HR=0.93, 95%CI,0.76-1.15, **Figure 8**), although there was statistically significant heterogeneity between trials (**Figure 8**).

### Bias analysis and sensitivity analysis

**Figure 9:**
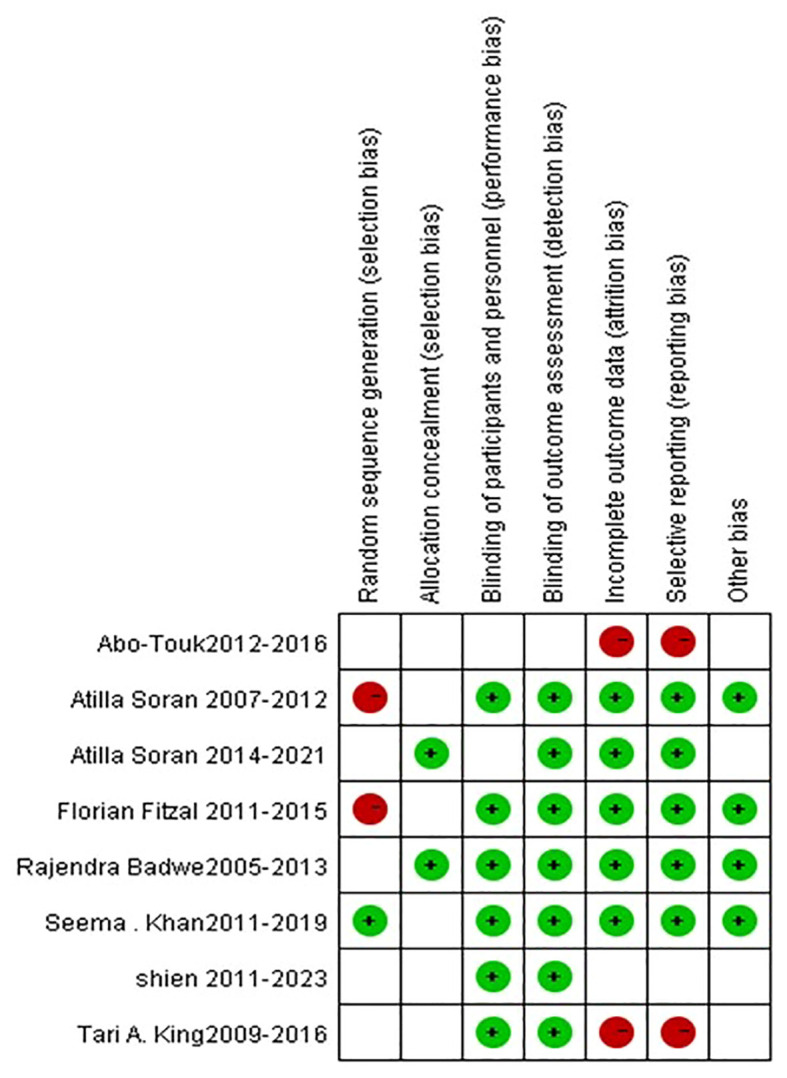
Risk plots for each study assessed using the Cochrane risk of bias tool.

**Figure 10:**
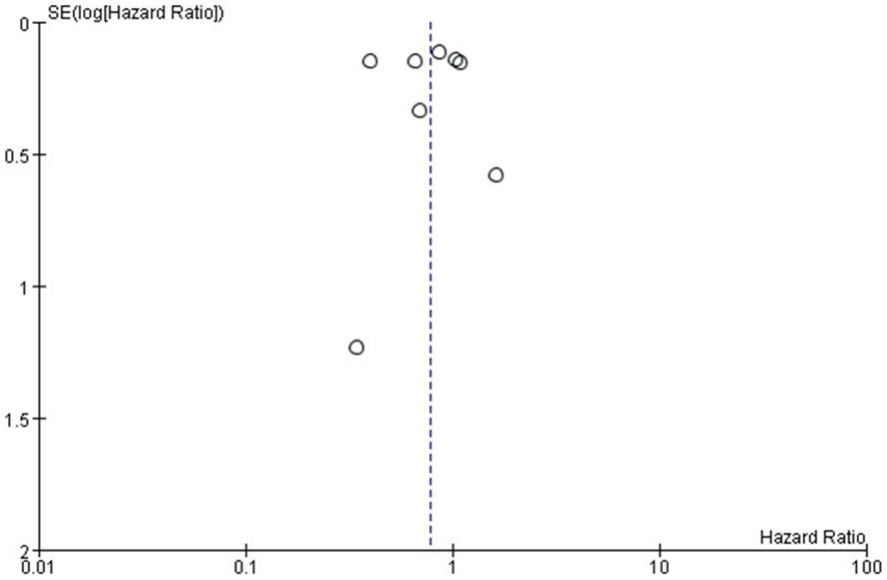
Funnel plot for statistical analysis of the studies included in the meta-analysis.

For all the eight studies included in the meta-analysis, the Cochrane Risk of Bias tool was used to assess the risk of bias (**Figure 9**). The funnel plot performed to assess the publication bias, as shown in **Figure 10**, indicated no publication bias being found in the included studies. The sensitivity analysis was conducted by deleting each study one by one and re-conducting the meta-analysis, as shown in **Table 2**. and The results of all the sensitivity analyses were consistent with the original analysis.

**Table 2:** The sensitivity analyses.

Analysis	Overall survival	I²	P
Primary Analysis HR 95%CI	0.78 [0.58, 1.05]	78%	0.1
Excluding Atilla Soran 2007-2012 HR 95%CI	0.80 [0.56, 1.15]	81%	0.23
Excluding Atilla Soran 2014-2021 HR 95%CI	0.88 [0.74, 1.06]	33%	0.19
Excluding Florian Fitzal 2011-2015 HR 95%CI	0.79 [0.57, 1.09]	81%	0.15
Excluding Rajendra Badwe 2005-2013 HR 95%CI	0.73 [0.52, 1.03]	78%	0.07
Excluding SeemaKhan 2011-2019 HR 95%CI	0.73 [0.52, 1.01]	77%	0.06
Excluding AboTouk 2012-2016 HR 95%CI	0.79 [0.58, 1.06]	81%	0.12
Excluding TA. King 2009-2016 HR 95%CI	0.75 [0.55, 1.01]	80%	0.06
Excluding Shien. T 2011-2023 HR 95%CI	0.76 [0.52, 1.12]	81%	0.16

## Discussion

Through analyzing the eight prospective studies (5 RCTs and 3 prospective studies) including more than 2000 patients, we found that the administration of locoregional surgery in primary tumors helped improving the local progression-free survival, other than the overall survival or distant progression-free survival in patients with *de novo* stage IV breast cancer. More interesting, based on the subgroup analyses on tumor subtype and metastatic site, the benefit of locoregional surgery in OS were found for patients with ER/ PR-positive diseases, bone-only metastasis. To our knowledge, this was the largest prospective study-based research to assess the impact of locoregional surgery in primary tumors on longterm OS.

It had been debatable whether or not the use of locoregional surgery in primary tumors, including breast-conserving surgery or mastectomy, axillary lymph dissection, or sentinel lymph node biopsy with or without radiotherapy, could help improve OS for patients with de novo stage IV breast cancer (28). Consequently, the indications of locoregional surgery in primary tumors were always considered as the followings: (1) symptomatic primary site (with the aim to control local symptoms); (2) progression of the primary tumor after controlled distant disease is controlled; (3) no evidence of disease except in the primary tumor (7, 8). In the current study, nearly half of the population included had underwent locoregional surgery in primary tumors. The result demonstrated indirectly the anxiety regarding the choice of therapeutic schedule in clinical work.

Survival of cancer patients, especially progression-free survival, distant progression-free survival, overall survival, important reliable, objective and easily accessible indicators, had been widely used in the evaluation and analysis of long-term prognosis of breast cancer patients. In our study, based on analysis of a large cohort of 2029 patients from 8 studies, the efficacy of locoregional surgery in primary tumors in prolonging the local progression-free survival was established, suggesting that locoregional surgery in primary tumors should not be omitting indiscriminately in patients with *de novo* stage IV breast cancer

As expectedly, in the current study, we found that locoregional surgery in primary tumors was associated with prolonging overall survival (OS) for patients with ER/PR positive breast cancer. The result was partly consistent to MF 07-01 trial, which concluded that after locoregional surgery and long-term follow-up, patients with *de novo* stage IV breast cancer had impvoed OS. The underlying reasons might be the low incidence of triple-negative and high incidence of ER/PR positive and isolated bone metastases rather than visceral metastases disease. However, due to no information on the association between surgical margin and outcomes, the choice of detailed surgical approaches for patients with de novo stage IV breast cancer remained ambiguous and vague. Some researchers found that total mastectomy and partial mastectomy with a definite negative margin were similar in the OS rate (28, 29). A meta- analysis (30) of 216 066 patients with de novo stage IV breast cancer also showed that both partial mastectomy and total mastectomy with negative margins can be used as local management options for patients with *de novo* stage IV breast cancer. Therefore, both breast- conserving surgery with negative margin and total mastectomy could be used as an alternative surgical procedure for patients with de novo stage IV breast cancer.

Moreover, this meta-analysis had certain limitations. First, there were differences in the experimental protocols of the included studies, such as the timing of surgery and different surgical methods, which could influence the overall survival of patients. Second, there was significant heterogeneity among the participants in the trials, and the different characteristics of the patients might affect the overall survival. Third, some trials did not show the complete outcome data or subgroup analyses, and some trials were showed in abstract form.

## Conclusion

Our study demonstrated that locoregional surgery in primary tumors was associated with improved OS for participants with ER/PR positive de novo stage IV breast cancer, and locoregional surgery in primary tumors could be worthy of clinical recommendation for patients with ER/PR positive de novo stage IV breast cancer. And, more prospective and randomized controlled trials of higher quality are needed to provide more convincing results.

## Data Availability

The original contributions presented in the study are included in the article/supplementary material. Further inquiries can be directed to the corresponding author.
